# Timing of TIPS for the management of portal vein thrombosis in liver cirrhosis

**DOI:** 10.2478/jtim-2023-0095

**Published:** 2023-12-20

**Authors:** Yong Lv, Yanglin Pan, Huahong Xie, Changbing Yang, Daiming Fan, Guohong Han

**Affiliations:** State Key Laboratory of Cancer Biology, National Clinical Research Center for Digestive Diseases and Xijing Hospital of Digestive Diseases, Fourth Military Medical University, Xi’an 710032, Shaanxi Province, China; Military Medical Innovation Center, Fourth Military Medical University, Xi’an 710032, Shaanxi Province, China; Department of Liver Diseases and Interventional Radiology, Digestive Diseases Hospital, Xi’an International Medical Center Hospital, Northwest University, Xi’an 710032, Shaanxi Province, China

## Introduction

Non-tumoral portal vein thrombosis (PVT) is the most common thrombotic event in patients with cirrhosis, with an annual incidence of 8%–12%.^[1]^ Decreased PV blood flow velocity (<15 cm/s) plays an important role in the development of PVT.^[2]^ The clinical manifestation of cirrhotic PVT is often mild or lacking. In many patients, diagnosis of PVT is set up by chance during regular outpatient visits for hepatocellular carcinoma screening or pretransplant workup. Several studies have reported that improvement or even spontaneous recanalization of PVT may occur in up to 70% of patients in the absence of any specific therapy.^[3,4]^ However, a progression of thrombus leading to complete occlusion of portal vein or extension to other splanchnic vessels has also been reported in 40%–70% of patients.^[3,4]^ The impact of PVT on outcome remains a matter of dispute. Nevertheless, it is clear that extensive and complete thrombus complicate the liver transplantation procedure and is associated with a higher post-transplantation mortality.^[5]^

## TIPS for the management of PVT in cirrhosis

In patients with cirrhosis, whether to treat PVT or not is still a debatable issue, given its controversial impact on the course and prognosis.^[1,6]^ Thus, in current or future liver transplantation candidates, therapy is recommended with aiming to recanalize the portal vein and to avoid thrombosis worsening and progression and thus achieve a physiological portal vein anastomoses and ensure portal flow to the graft. In the non-transplant patient with occlusive PVT, although it remains inconclusive, improving flow in the portal vein system should lead to easier control of portal hypertension, improvement in hepatic perfusion and prevention of cavernoma formation. In patients with compensated cirrhosis and partial PVT, given the higher likelihood of spontaneous recanalization, a conservative approach with close follow-up may be needed before starting treatment. In previous decades, PVT had been considered a contraindication to transjugular intrahepatic portosystemic shunt (TIPS) placement. However, since the first reports in the early 1990s, more experiences have been gained with TIPS in PVT cases, and it has been established as a valid therapeutic option.

### TIPS for PVT in cirrhosis: advantage and disadvantages

Placement of TIPS in patients with cirrhosis and PVT has several advantages. TIPS is highly effective in the recanalization of portal vein, removing clot and quickly reestablish physiologic flow in the portal vein in a matter of hours. Furthermore, TIPS reduces portal hypertension so has the additional benefits of reducing both ascites and variceal bleeding. Post-TIPS anticoagulation is generally not mandatory, since TIPS alone, by reconstructing a high velocity portal flow, was sufficient to maintain the long-term portal vein patency.^[7]^ Disadvantages to this therapy include the need for highly specialized and experienced proceduralists able to accomplish the procedure. In addition, hepatic encephalopathy (HE) is a major concern for TIPS. However, since portal perfusion is already diminished or abolished by the thrombosis before TIPS, the TIPS may not exert severe negative effects on liver function or HE.^[8]^

### TIPS for PVT in cirrhosis: technical aspects

In general, PVT makes the TIPS procedure more difficult because the intrahepatic branches of the portal vein may be occluded or stenosed, obstructing portal access by procedural catheters from the transjugular route.^[9]^ With the help of sonographic guidance, even very small intrahepatic branches can be detected and punctured successfully.^[10]^ A percutaneous transhepatic and trans-splenic routes can also be adopted to allow better visualization of the portal vein before transjugular puncture.^[10]^ In the case of chronic complete/occlusive PVT, guidewire crossing of the occluded segment may be difficult even though access of intrahepatic portal branch was achieved. A percutaneous transhepatic or trans-splenic approach could provide a better angle for endovascular manipulations and an easier handle for probing the occlusion. If recanalization of the obliterated portal trunk was impossible, TIPS could be selectively inserted in a large collateral vein.^[11,12]^ In case of very extensive portal vein thrombus, stent deployment is also more challenging if the thrombus extended into the superior mesenteric vein (SMV). Local mechanical thrombectomy combined with pharmacological thrombolysis during the TIPS procedure may help to restore normal blood flow.^[13]^ Nevertheless, in patients with complete occlusion of both splenic and mesenteric veins, TIPS placement is not recommended because inadequate blood flow into shunt may cause an invalid TIPS insertion.^[10]^

### TIPS for PVT in cirrhosis: clinical results

A series of studies has reported the efficacy and safety of TIPS for the treatment of PVT. In a recent meta-analysis of 13 studies including 399 patients (92% cirrhosis; PVT: complete 46%, chronic 87%, portal cavernoma 15%), TIPS was technically feasible in 95% of cases, resulting in a portal vein recanalization rate of 79% at 12 months.^[14]^ The rates of major complications were 10%. Re-thrombosis and shunt dysfunction was not frequent after TIPS.^[14]^ The response to TIPS was associated with the age and extension of the thrombus, the presence of varices, and the degree of thrombosis in the portal trunk. The rebleeding rates were very low after successful TIPS and significantly higher when compared to patients with unsuccessful intervention.^[9,10,15]^ In patients with PVT complicated with portal hypertensive complications, a recent randomized controlled trial comparing TIPS with endoscopic band ligation and propranolol with TIPS for secondary prophylaxis of variceal bleeding in patients in patients with cirrhosis and PVT showed that TIPS was more effective than medical and endoscopic therapy without an increase in the risk of HE, leading to a recanalization rate of 95%.^[13]^ In patients with chronic complete occlusive PVT or those who have already developed portal cavernoma, TIPS placement is also a big challenge. Nevertheless, the introduction of transplenic and/or transhepatic approach is changing this scenario. In a series of 61 patients with cirrhosis and chronic PVT, though a transplenic and/or transhepatic approach, technical success rate was up to 98%.^[16]^ The patency of the portal vein and TIPS was maintained in 92% of patients with median follow-up of 19 months. Posttransplant outcomes were excellent with 24 patients (39%) undergoing transplantation, 96% of whom received an end-to-end anastomosis.^[16]^

## Timing of TIPS for PVT in cirrhosis

Tinging of TIPS placement is based on the chance of response to anticoagulation, underlying the stage of cirrhosis, presence or complications of portal hypertension, and eventual candidacy to liver transplant.

### Patients without symptomatic portal hypertension

In patients without symptomatic portal hypertension, anticoagulation is the primary treatment of acute PVT.^[1,6]^ The advantages of anticoagulation include that it is widely available, does not require specific procedural techniques, can be started and stopped as needed, has low rates of specific complications, and does not permanently alter anatomy. Observational nonrandomized studies have shown that after anticoagulation therapy, PVT improvement is observed in 60%–100% of patients without increasing the risk of bleeding.^[17,18]^ The timing of TIPS in such cases is the second-line choice for patients not response to anticoagulation. Although the treatment failure and optimal duration of anticoagulation has not well defined, the decision for the TIPS in this setting should be made early to increase its success rate and efficacy. It should be pointed out that in those without portal hypertensive complications, the aim of the TIPS is to improve portal blood flow velocity but not to normalize portal hypertension. Thus, stents with small diameter (6–8 mm) are preferred to avoid excessive shutting.

### Patients with complications of portal hypertension

Patients with PVT who have complications of portal hypertension that are refractory to medical treatment should be evaluated for TIPS placement. The simultaneous presence of complications of portal hypertension that could potentially limit or delay the use of anticoagulants. In addition, side-effects of anticoagulation are unneglectable in patients with decompensated cirrhosis, in particular in those with platelet counts less than 50 × 109/L, encompassing higher risk of variceal bleedings and cerebral bleeding.^[6]^ Additional issues arguing against anticoagulation are the probably limited compliance of long-term low molecular weight heparin injection and the problem with monitoring of vitamin K antagonists.^[18]^ By comparison, TIPS in addition to increased rates of portal vein recanalization reduces portal hypertension so has the additional benefits of reducing both ascites and variceal bleeding. Furthermore, studies have showed that preemptive TIPS (placed within 72 h) compared to medical and endoscopic therapies was associated with improved survival in selected patients with cirrhosis and acute variceal bleeding (Child-Pugh class C 10-13 points or Child-Pugh class B 8-9 points with active bleeding at initial endoscopy despite vasoactive drug treatment or hepatic venous pressure gradient > 20 mmHg at the time of bleeding).^[19,20]^ Thus, the TIPS placement is justified in patients with PVT and complications of portal hypertension (variceal bleedings or tense ascites) without prior use of anticoagulants, and should be used as soon as possible.

### Patients with chronic complete occlusive PVT awaiting liver transplantation

In the liver transplant candidates, establishment of a patent main portal vein allows for end-to-end anastomosis of the donor to recipient portal veins. In chronic complete occlusive thrombosis, the response of anticoagulation is very limited, thus questioning its use.^[18]^ By contrast, a transplenic or transhepatic approach, known as portal vein recanalization-TIPS, was shown to improve technical success to over 90% in the most recent series (range 75–100%).^[10,16]^ In these procedures, portal vein recanalization is performed by angioplasty/stenting with subsequent TIPS insertion to ensure the outflow of the system.^[10,16]^ As such, in experienced centers TIPS can be considered as first-line therapy for patients with chronic occlusive PVT who are in need of liver transplant.

## Summary

PVT is a frequent complication in patients with liver cirrhosis. The therapeutic decision for the use of TIPS for the management of PVT is often determined by the age and extent of thrombosis, the presence of portal hypertension, and liver transplant eligibility. TIPS can be utilized for cirrhotic patients in whom thrombosis persists or progresses despite optimal anticoagulation therapy, in those who present with complications of portal hypertension, and those with chronic complete occlusive PVT awaiting liver transplantation.
